# Supporting shared hypothesis testing in the biomedical domain

**DOI:** 10.1186/s13326-018-0177-x

**Published:** 2018-02-08

**Authors:** Asan Agibetov, Ernesto Jiménez-Ruiz, Marta Ondrésik, Alessandro Solimando, Imon Banerjee, Giovanna Guerrini, Chiara E. Catalano, Joaquim M. Oliveira, Giuseppe Patanè, Rui L. Reis, Michela Spagnuolo

**Affiliations:** 1 Italian National Research Council, Via De Marini 6, Genoa, 16149 Italy; 2 University of Oslo, Oslo, Norway; 3 3B’s Research Group, Biomaterials, Biodegradables and Biomimetics, Headquarters of the European Institute of Excellence on Tissue Engineering and Regenerative Medicine, University of Minho, Caldas das Taipas, Portugal; 4 ICVS/3B’s - PT Government Associate Laboratory, Braga/Guimarães, Portugal; 5 University of Genoa, Genoa, Italy; 6 Center for Medical Statistics, Informatics, and Intelligent Systems, Institute for Artificial Intelligence and Decision Support, Medical University of Vienna, Spitalgasse 23, Vienna, 1090 Austria; 7 Department of Biomedical Data Science, Stanford University School of Medicine, Stanford, 94305 California USA

**Keywords:** Biomedical ontology, Ontology mapings, Network analysis, Hypothesis testing, Incomplete knowledge

## Abstract

**Background:**

Pathogenesis of inflammatory diseases can be tracked by studying the causality relationships among the factors contributing to its development. We could, for instance, hypothesize on the connections of the pathogenesis outcomes to the observed conditions. And to prove such causal hypotheses we would need to have the full understanding of the causal relationships, and we would have to provide all the necessary evidences to support our claims. In practice, however, we might not possess all the background knowledge on the causality relationships, and we might be unable to collect all the evidence to prove our hypotheses.

**Results:**

In this work we propose a methodology for the translation of biological knowledge on causality relationships of biological processes and their effects on conditions to a computational framework for hypothesis testing. The methodology consists of two main points: hypothesis graph construction from the formalization of the background knowledge on causality relationships, and confidence measurement in a causality hypothesis as a normalized weighted path computation in the hypothesis graph. In this framework, we can simulate collection of evidences and assess confidence in a causality hypothesis by measuring it proportionally to the amount of available knowledge and collected evidences.

**Conclusions:**

We evaluate our methodology on a hypothesis graph that represents both contributing factors which may cause cartilage degradation and the factors which might be caused by the cartilage degradation during osteoarthritis. Hypothesis graph construction has proven to be robust to the addition of potentially contradictory information on the simultaneously positive and negative effects. The obtained confidence measures for the specific causality hypotheses have been validated by our domain experts, and, correspond closely to their subjective assessments of confidences in investigated hypotheses. Overall, our methodology for a shared hypothesis testing framework exhibits important properties that researchers will find useful in literature review for their experimental studies, planning and prioritizing evidence collection acquisition procedures, and testing their hypotheses with different depths of knowledge on causal dependencies of biological processes and their effects on the observed conditions.

## Background

Diseases and pathologies may be evidenced across multiple biological scales (e.g., cellular, molecular, organic, behavioural) as a set of factors, linked among each other via causal relationships, which constitute the multi-scale pathological cascade reactions. To study the underlying causation mechanism of a certain disease, life science researchers rely on various sources, such as (i) current knowledge (e.g. previously published studies from the field), (ii) their data deduced from empirical analysis of laboratory experiments (e.g., gene analysis, immuno-assays, cell viability assays, histology) or other tests (i.e. mechanical tests, imaging, gait analysis), as well as on (iii) consultations with other fields (i.e. related research areas, hospitals). To effectively make and test (prove or reject) a causality hypothesis life science research studies face two challenges: i) the information used in research processes comes from various sources and is heterogeneous, which makes it hard to organize, analyze, and assess their relevance in the overall disease process, ii) researchers from different fields (i.e. molecular biologist, mechanobiologist, orthopaedists etc.) investigate the same pathological event from different aspects (biological scales), and might not be aware of the overlaps and the impact of their individual findings in a joint venture of understanding causality mechanisms of pathologies and diseases.

To better convey the idea of causality hypothesis testing we will focus on knee articular cartilage degeneration during the onset of osteoarthritis (OA) to present our use-case scenario. OA is a joint degenerative disease and can be caused due to several factors, such as genetic predisposition, joint overuse, previous injury to the joint. The effect of these factors is hallmarked with a complete joint breakdown and dysfunction, causing a lot of pain [1, 2]. Based on common knowledge, performed experiments, and diagnosis the causality relation of certain factors to the development of OA might have different degrees of confidence. On the one hand, the degeneration of cartilage, synovial thickening, osteophyte formation and joint space narrowing, are known to be as the most marked features of OA [3–6]. On the other hand, for some factors we may have lower degrees of confidence in their causality relationship to OA. For instance, while being common in patients with OA, the exact causality relation of inflammation to OA is not completely understood [7, 8]. To handle such scenarios of causality hypothesis testing, we propose to translate what we observe in the biology into a computational framework, which supports the researchers in their hypothesis testing. In such a framework we systematically translate our background knowledge on causality relationships into the representations suitable for the computation, and we quantify confidences in our hypothesis with respect to the amount of evidences that we can supply to the framework.

### Hypothesis testing

**Fig. 1:**
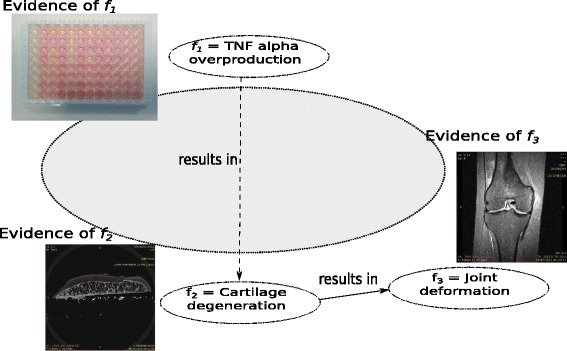
Causality hypothesis of TNF alpha overproduction leading to cartilage degeneration and provoking joint deformation

Schematically, the causality relationships between the factors of diseases can be represented as directed causality networks *H*_0…*n*_, where factors *f*_*i*_ are represented as nodes and the causality relationships as arcs (*f*_*i*_, *f*_*j*_). For instance, our hypothesis *H*_0_ can state that inflammation contributes to the development of OA, where the inflammation is the cause of biological processes which lead to cartilage degradation (factor *f*_2_, Fig. 1) and finally manifest in joint deformation condition (factor *f*_3_, Fig. 1). To prove such a causality hypothesis we need to evidence the instances of all the participating factors. For example, the factors *f*_2_, *f*_3_ are evidenced as the results of diagnosis of OA done by radiologists and orthopaedists using imaging techniques (i.e. magnetic resonance-MRI, X-ray). By studying the literature we can discover that the inflammation can be characterized by the detection of high levels of pro-inflammatory factors in the synovial cavity, and in particular tumor necrosis factor alpha (TNF *α*) (factor *f*_1_ in Fig. 1), was demonstrated to be present in excess during OA [9]. A justification or evidence for the factor *f*_1_ (evidence of *f*_1_ in Fig. 1) can be obtained with molecular biological techniques screening the biomarkers of the synovial fluid. Given our knowledge of the participating biological processes (hypothesis *H*_0_) and the supporting evidences (evidences for factors *f*_1_, *f*_2_, *f*_3_) we have a certain level of confidence that the synovial inflammation has been the cause of the development of OA. However, is our hypothesis *H*_0_ complete enough, and are the evidences for factors (*f*_1_, *f*_2_, *f*_3_) enough to support our hypothesis? Have we missed other factors? Have we been complete enough in our characterization of all the participating factors which support the hypothesis that the synovial inflammation has been the cause of cartilage degradation? Is the joint deformation the only consequence of such a pathological cascaded of reactions?

**Fig. 2:**
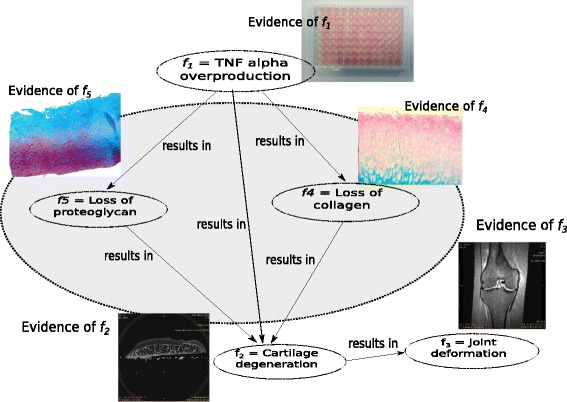
Refined causality hypothesis of pro-inflammatory factors leading to loss of building blocks of articular cartilage – collagen and proteoglycan –, which in turn lead to cartilage degeneration and provoking joint deformation

Studying further the causality mechanism of OA, we can refine our initial hypothesis *H*_0_. In particular, cellular biological studies observed that TNF *α* facilitates the catabolic processes of the chondrocytes, including the production of matrix metalloproteinases (MMPs), and the production of aggrecanases (members of the ADAMTs family) [10, 11]. The MMPs, especially MMP-13 and aggrecanases are proteases responsible for the degradation of collagen macromolecules and proteoglycans respectively, as evidenced in literature [12]. Collagens and proteoglycans are the main building blocks of articular cartilage. Accordingly, the excess of TNF *α* in the joint space can be associated to the disruption of biochemical balance in the cartilage. Factors: Loss of collagen and proteoglycan molecules (factors *f*_4_,*f*_5_ in Fig. 2), are caused by the action of matrix degrading proteases, and can be attached to higher scales in the OA processes, such as the mechanical functioning of cartilage. These factors can be evidenced on the tissue level by histology and immuno-histochemistry (evidences of *f*_4_, *f*_5_ in Fig. 2). Collaborations with mechano-biological fields allow the detection of the changes in cartilage mechanical properties due to the effect of high levels of MMPs and aggrecanses [13, 14]. It has been shown previously that once the cartilage suffers collagen loss, it is no longer able to withstand the mechanical forces in the knee [15, 16]. Consequently, the cartilage, the trabecular bone beneath it, and all surrounding tissue components suffer damage, which can be evidenced by imaging [17, 18]. Damage to the joint components, will cause pain, joint deformation and loss of function, which is a subject of behavioural scales and can be evidenced by gait analysis [19].

The relationship between inflammation and OA is even more complex, than the example brought above. Nonetheless, collaborations among medical doctors and bench researchers of various fields can reveal the connections between molecular evidence and those observed on organ scale. Accordingly, we can refine our hypothesis by adding new causal relationships.

### Shared hypothesis testing framework

In this work we propose a methodology for the translation of biological knowledge on causality relationships of biological processes and their effects on conditions to a computational framework for hypothesis testing. The methodology consists of two main points: hypothesis graph construction from the formalization of the background knowledge on causality relationships, and confidence measurement in a causality hypothesis as a normalized weighted path computation in the hypothesis graph. In this framework, we can simulate collection of evidences and assess confidence in a causality hypothesis by measuring it proportionally to the amount of available knowledge and collected evidences. We evaluate our method on an example causality hypothesis of factors which cause and, in turn, may be caused by cartilage degeneration during osteoarthritis. The results of the evaluation and the feedback from the domain experts allow us to conclude that our methodology may simulate the execution of evidence collection, and can be used as a means of measuring the confidence in a causality hypothesis with respect to the amount of knowledge on causality relationships among participating factors. Such simulation supports the researchers in the planning and in the prioritization of their next studies by identifying important factors in a causality hypothesis. Our methodology demonstrates robustness towards the addition of potentially inconsistent knowledge by separately representing opposite causality possibilities for complementary biological scenarios.

We would like to emphasize that the contribution of this work is the methodology to extract the causality information from the input ontologies into a hypothesis graph, and perform hypothesis testing on the obtained hypothesis graph. The ontologies and the ontology mappings discussed and provided are created together with the domain experts, and in the context of this work are only meant to serve as proof of concept.

### Related work

To the best of our knowledge the proposed methodology to test a causality hypothesis in a collaborative setting with respect to the amount of knowledge available for the framework does not have an equivalent methodology or an implemented system to test against, in its entirety. However, once decomposed, our methodology can be compared on specific steps and modelling choices.

**Formalization of background knowledge on a causal hypothesis as ontologies.** Our methodology for causality hypothesis testing relies on the formalization of the background knowledge on a hypothesis with ontologies. Indeed, to facilitate knowledge sharing and increase understanding of the method in use, it is common to employ already existing ontologies that are well agreed on in the biomedical community (e.g., Gene Ontology [20]). The most widely used ontology modeling language is the (OWL 2) [21], based on formal logic [22]. The main advantage of using logic over alternative representation mechanisms is that logic provides an unambiguous meaning to ontologies. We assume that the input ontologies to our framework focus on (biological) processes and findings (i.e., laboratory tests) that are or may be linked via a causality relationship, and other (material) entities that (actively or passively) participate in the process or finding. In this work we assume that the input ontologies follow good practices and relevant ontology classes are either subsumed by or annotated with, for example, the concept Biological_process (key concept in the *Gene Ontology* [20]) or Finding (e.g., common semantic type in the *UMLS semantic network* [23]). We expect the following (object) properties or its potential subproperties as source for causality relationships: causes, results in, regulates, positively regulates, negatively regulates, increases levels of and decreases levels of. Most of these properties are available in the *Relations ontology* [24] and are extensively used in biomedical ontologies. We reuse the domain independent categories *Continuant* and *Occurrent*, which are commonly used in the literature (e.g., River Flow Model of Diseases (RFM) [25]) and in upper ontologies (e.g., DOLCE [26] and BFO [27]). For example, processes and findings are typically classified as *occurrents*, while material entities as *continuants*.

**Graph projection of OWL ontologies.** The hypothesis graph construction heavily relies on the graph projection of OWL ontologies. This procedure, at its core, transforms an OWL ontology into its graph representation, by studying the axiomatic structure of the ontology and identifying nodes and edges (arcs) of its equivalent graph representation. Implicitly, Lembo et al. [28] use graph projections of OWL QL to propose ontology classification algorithm, which transforms OWL QL ontologies into directed graphs, and computes subsumption relations via transitive closure computation. Analogously, Seidenberg et al. [29] use graph representation of ontologies to propose a segmentation algorithm based on subgraph extraction procedure. Some of the proposed methodologies for graph projection of OWL ontologies draw their inspirations from Social Network Analysis (SNA) [30] for the representation of the encoded semantic information in an OWL ontology. SNA is the process of investigating social structures of connected information/knowledge entities through the use of network and graph theories. SNA techniques application to ontology analysis has been pioneered by Hoser et al. [31], where standards in SNA community graph metrics based on: node degree, node betweenness and on eigenanalysis of the adjacency matrix, were used to study properties of ontologies. The connection between SNA and ontology analysis have also been studied in a highly cited paper by Mika [32], bridging Social Networks and Semantics. Network partitioning algorithms have been used by Stuckenschmidt et al. [33] to identify islands of ontology, a notion comparable to a module of ontology (as used by the graph-based modular extraction community), with the applications to Visual Analytics. Grontocrawler [34] transforms OWL-EL [35] ontologies into networks by defining a rule-based edge production procedure, which takes into account existential and values restrictions on object relations. Formal treatment of rule-based graph projection procedures and their connection to the logical entailment problem for OWL 2 ontologies have been recently proposed [36–38]. In our work we use Grontocrawler [34] for graph-based ontology projection, enriched with the projection of advanced OWL 2 axioms, as suggested in Soylu et al. [38].

**Rule-based reasoning with incomplete knowledge in the biomedical domain.** Similarly to previous works [39, 40], we focus on graph-based reasoning with incomplete knowledge, by analyzing OWL ontologies, to support researchers in the biomedical domain. In particular, Larson et al. [39] propose a method for rule-based reasoning with a multi-scale neuroanatomical ontology, where the authors conclude that OWL is an important technology for merging disparate data and performing multi-scale reasoning. They demonstrate how OWL-based ontologies and rule-based reasoning help infer novel facts about brain connectivity at large scale from the existence of synapses at a micro scale. Oberkampf et al. [40] propose a methodology for interpreting patient clinical data (medical images and reports), semantically annotated by concepts from large medical ontologies. They introduce an ontology containing lymphoma-related diseases and symptoms as well as their relations and use it to infer likely diseases of patients based on annotations.

In contrast to Larson et al. [39] our graph-based reasoning method relies on network analysis of the final hypothesis graph, which presents an advantage of a full overview of all possible conclusions with the quantification of the confidence measure induced by the number of evidences that have been collected and the final topology of the hypothesis graph. Oberkampf et al. [40] focus on the problem of inferring likely diseases in the presence of patient-specific evidences, represented as symptoms, and the similarity of the diseases is then ranked based on their distances to the symptoms. The focus of our work and the methodology are different. We tailor our causality hypotheses to a single diseases and study causality relationships among the factors, the findings obtained with our methodology may have impact not only in the clinical, patient-specific setting, but can be used in general research. Technically, our methodology for graph projections employs a rich set of OWL 2 axioms, and go beyond the usual taxonomical relationships which can be extracted from the ontologies.

**Probabilistic methodologies for reasoning with incomplete knowledge and causality inference, with applications in the biomedical domain.** In a more general setting, not necessarily connected to the biomedical domain, there are examples of general theoretical frameworks which marry formal methods (e.g., First-Order Logic) and probabilistic models (e.g., stochastic processes) [41–43]. Application of those methodologies in biology is studied in Ciocchetta et al. [44] who tune the Stochastic Process Algebra language PEPA [43] to model biological pathways and complex biological networks, involving stochastic processes. This line of works bridge “uncertainty” and “formal methods” for general frameworks for reasoning with incomplete knowledge in biology, and differently with our methodology is not compatible with OWL ontologies, and thus cannot benefit from OWL reasoning tasks (e.g., classification, alignment).

Our work is perhaps similar in spirit to that of Pearl et al. [45, 46], where the authors advocate for a paradigmatic shift that must be undertaken in moving from traditional statistical analysis to causal analysis of multivariate data [45, 46]. Pearl et al. propose a formal treatment and a unified methodology for the graphical representation of joint probability distributions along with rules for inferring causality directly from such graphical representations. In particular, the directed graphs are introduced as a compact way of representing conditional independence restrictions for complex multidimensional probability distributions. In contrast, in our work we do not stress the existence of joint probability distributions between the factors of a hypothesis. Rather, we rely on expert knowledge of causality relationship between the factors, already known to the community, such as knowledge graphs which can be obtained from literature sources, and/or can be formalized in an OWL ontology by the domain experts.

## Methods

**Fig. 3:**
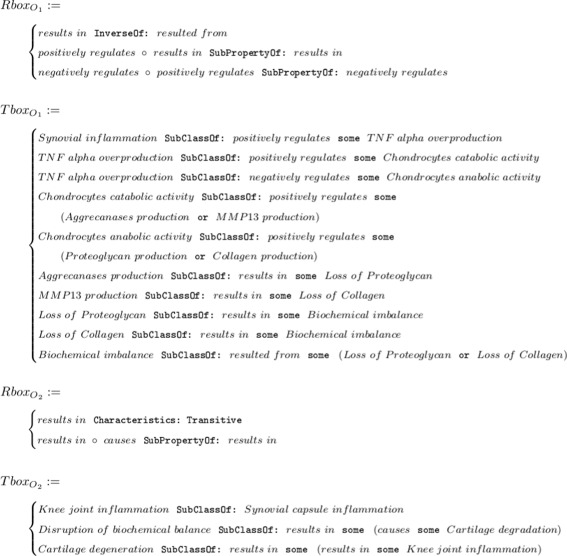
Formalization of knowledge on OA pathogenesis processes

Herein we assume that there exists a universal causality hypothesis *H* that can be represented as a network of factors with causality relationships, which we call a *hypothesis graph*. The background knowledge on the hypothesis graph *H* is formalized in an ontology *O*, which, for instance, may define factors as biological processes and conditions, and the causality relationships may indicate the connections between them. Moreover, we assume that different experts formalize the background knowledge on *H* in ontologies *O*_*i*=1…*n*_, such that each *O*_*i*_ highlights a certain subpart of this hypothesis graph *H*. Consider $O_{1} = \langle Rbox_{O_{1}}\phantom {\dot {i}\!}$, $Tbox_{O_{1}} \rangle $, $\phantom {\dot {i}\!}O_{2} = \langle Rbox_{O_{2}}$, $Tbox_{O_{2}} \rangle $ in Fig. 3, the examples of formalization of the the causality relationships among biological processes that participate in OA pathogenesis, from two different points of view.

**Fig. 4:**
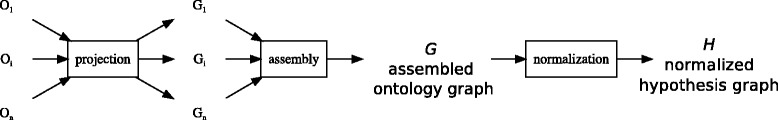
Our methodology defines a pipeline to transform background knowledge into a hypothesis graph via sequential application of processing steps: *projection* of input *O*_*i*_ ontologies into ontology graphs *G*_*i*_, *assembly* of an ontology graph *G* with input ontology mappings *m*_*i*_, normalization of the ontology graph *G* into a final hypothesis graph *H*

The overlaps among the ontologies *O*_*i*_ may or may not exist and, as the number of ontologies increases, we assume that it is possible to assemble (align) these ontologies. The assembled ontology $\bigcup _{i}^{n} O_{i} = O$ represents the iteratively gathered and formalized biological and biomedical knowledge on the hypothesis graph *H*. Finally, the causality hypothesis graph *H* – the network of factors interconnected with causality relationships – can be extracted from the assembled ontology *O* at any given point in time *t*_*i*_ ($H_{t_{0}}, \ldots, H_{t_{n}}$). As a consequence, the *shape* of the causality hypothesis $H_{t_{i}}$ depends on the amount of background knowledge formalized in *O* at *t*_*i*_. Finally, the hypothesis graph construction from ontologies is performed in a three-step process: (1) projection of OWL 2 ontologies *O*_1_,…,*O*_*n*_ into ontology graphs *G*_1_,…,*G*_*n*_, (2) assembly of the ontology graph *G* from *G*_1_,…,*G*_*n*_, and (3) normalization of the graph *G* to obtain the hypothesis graph *H* (Fig. 4).

### Graph-based ontology projections

The nodes of the ontology-graph are unary predicates and edges are labelled with possible relations between such elements, that is, binary predicates. The key property of this ontology-graph is that every *X*-labelled edge *e*=(*v*,*w*) is justified by one or more axioms entailed by the ontology which “semantically relates” *v* to *w* via *X*. For example, edges *e* of the form $A \xrightarrow {broader} B \ $ are justified by the OWL 2 axiom: *B* SubClassOf: *A*. We rely on the OWL 2 reasoner HermiT [47] to build the ontology graph (e.g., extraction of classification) to consider both explicit and implicit knowledge defined in the ontology *O*. In the following, *A*,*A*_*sup*_,*A*_*sub*_,*B*,*B*_*i*_ represent classes, while *R*,*S*,*S*_*i*_,*R*^−^ represent object properties. Edges *e* of the form $A \xrightarrow {R} B \ $ are justified by the following OWL 2 axioms:

(i) ‘ *A* SubClassOf: *R* restriction *B*’, where *restriction* is one of the following: *some* (existential restriction), *only* (universal restriction), *min x* (minimum cardinality), *max x* (maximum cardinality) and *exactly x* (exact cardinality).
  Note that axioms with an union of classes in the restriction (e.g. ‘ *A* SubClassOf: *R* restriction
  *B*_1_ or … or *B*_*n*_’) or an intersection of classes in the restriction (e.g. ‘ *A* SubClassOf: *R* restriction
  *B*_1_ and … and *B*_*n*_’) also justify edges of the form $A \xrightarrow {R} B_{i} \ $ with 1≤*i*≤*n*.
(ii) Nesting (one level) with the same object property:
  ‘ *A* SubClassOf: *R* restriction (*R* restriction *B*)’, being *R* transitive.
(iii) Nesting (one level) with different properties:
  ‘ *A* SubClassOf: *R* restriction (*S* restriction *B*)’, and the role chain axiom of the form: ‘ *R* ∘ *S* SubPropertyOf: *R*’.
(iv) A combination of range and domain axioms of the form: ‘ *R* Domain: *A*’ and ‘ *R* Range: *B*’.
(v) Role chain axiom of the form: ‘ *S*_0_ ∘⋯∘ *S*_*n*_
  SubPropertyOf: *R*’ when the ontology graph already includes the edges $A \xrightarrow {S_{0}} C_{1} \dots C_{n} \xrightarrow {S_{n}} B$.
(vi) ‘ *R* InverseOf: *R*^−^’ when the ontology graph already includes the edge $B \xrightarrow {R^{-}} A$.
(vii) Top-down propagation of restrictions:
  ‘ *A* SubClassOf: *A*_*sup*_’ when the ontology graph already includes the edge $A_{sup} \xrightarrow {R} B$.
(viii) Entailment among restrictions:
  ‘ *B*_*sub*_ SubClassOf: *B*’ when the ontology graph already includes the edge $A \xrightarrow {R} B_{sub}$.

### Assembly of ontology graphs

The ontologies formalizing the hypothesis graph may be created by different group of experts with different modelling (e.g., defining relationships between occurrents, or between ocurrents and continuants) and naming conventions. For example, a group may use the concept Cartilage degradation (occurrent) from SNOMED-CT [48] while another may prefer to use the concept negative regulation of cartilage development (occurrent) from the GO [20]. Furthermore, other groups would rather use the concept Cartilage (continuant) and push the semantics of *degradation* into the ontology property.

Ontology alignment will enable the integration and assembly of the (sub-)ontology graphs in a larger ontology graph. An ontology alignment is composed by a set of ontology mappings. An ontology mapping *m* between two concepts *C*_1_,*C*_2_ from the vocabulary of two different ontologies *O*_1_,*O*_2_ can be defined as follows: *m*=〈*C*_1_,*C*_2_,*r*〉, where *r* is the relation between *C*_1_ and *C*_2_ and, using SKOS vocabulary, it can be of one of the following types: *skos:exactMatch*, *skos:closeMatch*, *skos:relatedMatch*, *skos:narrowMatch* or *skos:broadMatch*.

Mappings to guide the assembly (i.e., link factors from different hypothesis) can be discovered in online resources like UMLS Metathesaurus [49] and BioPortal [50, 51], or using state of the art ontology alignment systems like LogMap [52] and AML [53]. Mappings in UMLS Metathesaurus or BioPortal typically represent correspondences of the type *skos:exactMatch* and *skos:closeMatch*,^1^ while the output provided by automatic systems will typically provided mappings of diverse type and quality.

If a mapping exists to link two factors *f*_1_ and $f_{1}^{'}$ from two different (sub-)ontology graphs, then these two factors are merged into one. The weight of the merged factor will be according to the type of the ontology mapping. In our setting, we assume the following weight values *w* (ranging from 0 to 1) depending on the mapping type: (1) *skos:exactMatch* mappings are associated with a weight value 1.0, (2) *skos:closeMatch* mappings with 0.75, while (3) *skos:relatedMatch*, *skos:narrowMatch* and *skos:broadMatch* with a weight of 0.5. The weight associated to each (merged) factor will play a key role in our methodology for confidence measurement in a hypothesis.

### Normalization of the assembled graph

The final step of hypothesis graph construction is the *normalization* of the assembled hypothesis graph, which pushes the rich semantics of causality relationships (e.g., edges of the type $A \xrightarrow {R} B \ $) into, possibly newly created, nodes. Generally speaking, the normalization procedure leads to a simplified representation of all the available facts on causality relationships as a directed graph with specific constraints on the types of nodes and edges. Specifically, we aim to build a 1-mode network where all the nodes represent the same fundamental metaphysical type (occurrent), and all the edges represent the simplified causality relationship defined between two occurrents. This is necessary because the general graph projection step of our pipeline might produce semantic networks of concepts where the concepts and the edges may have different types. For instance, the ontology graph may contain edges representing causality relationships involving both an occurrent and a continuant – two fundamentally different metaphysical types of concepts. Additionally, the semantics of causality relations may reflect complementary effect when we consider causal chains in the hypothesis graph, for instance *negative* and *positive* regulations of biological processes. The hypothesis graph normalization consists in iterative rewriting of the graph, where we filter all edges and rewrite them according to the following patterns:

(i) $Occurrent \xrightarrow {R} Occurrent \ $ where *R* represent the property *results* *in* or *causes* justifies the edge in the hypothesis graph *Occurrent*↦*Occurrent*. For example, if the ontology contains the axiom, ‘Chondrocyte catabolism SubClassOf: results in some Collagen degradation’ the ontology graph will include the edge Chondrocytes catabolism$\xrightarrow {results\, in}$Collagen degradation and the hypothesis graph will contain the causality relationship Chondrocytes catabolism ↦Collagen degradation.
(ii) $Occurrent \xrightarrow {R} Occurrent \ $ where *R* represent the property positively regulates or negatively regulates. In this case the positive or negative semantics of the property are pushed to a fresh *ocurrent* concept. For example, if the ontology projection contains the edge Chondrocytes anabolism$\xrightarrow {positively\, regulates}$Collagen production, we will add the causal relationship Chondrocyte anabolism ↦Positive regulation of Collagen production.
(iii) $Occurrent \xrightarrow {R} Continuant \ $ where *R* represent the property positively regulates, negatively regulates, increases levels of or decreases levels of. For example if the ontology graph includes the edge TNF alpha overproduction$\xrightarrow {decreases\, levels\, of}$Collagen the hypothesis graph will include the fresh term Decreased levels of Collagen (or Loss of Collagen) and the causal relationship TNF alpha overproduction ↦Decreased levels of Collagen.

**Fig. 5:**
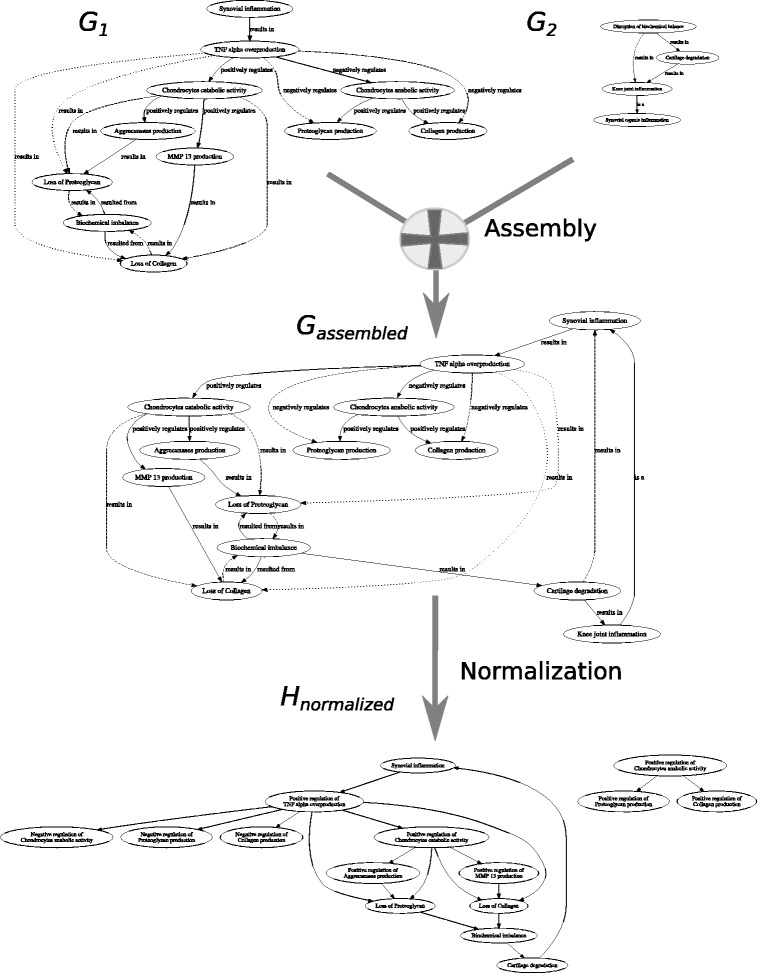
Schematic representation of the three-step pipeline for the hypothesis graph *H* creation from the two input ontologies *O*_1_,*O*_2_: i) use graph projection rules to transform each ontology *O*_*i*_ into its graph representation, ii) assemble the hypothesis graph *H* from two ontology graphs by merging concepts for which we have ontology mappings *m*_*i*_, and finally iii) normalize the hypothesis graph *H* by extracting only the relevant information of causality relationships among the occurrents

In Fig. 5 we illustrate the whole pipeline of constructing a hypothesis graph *H* from the two input ontologies *O*_1_,*O*_2_, defined in Fig. 3. The two ontology graphs *G*_1_,*G*_2_ represent the individual extent of background knowledge of the two specialists on causality relationships of factors between synovial inflammation and cartilage degradation (obtained by projecting ontologies *O*_1_,*O*_2_). The assembly of the graphs takes as input the ontology mappings *m*_1_ and *m*_2_ (see Table 1), which have been manually created by the domain experts, to merge the graphs *G*_1_,*G*_2_. Overall, the graph projection and the graph assembly steps of the pipeline work in couple to entail new causal links among the factors, which we represent in the assembled graph *G*. For instance, once we align the two graphs we entail the circular causality relationship, which states that Synovial inflammation may be, simultaneously, the cause and the effect of Cartilage degradation. Notice that before the alignment the two specialists were not aware of this circular relationship. The normalization of the assembled graph *G* splits the two biological scenarios of chondrocytes’ anabolic and catabolic activities, such that the resulting hypothesis graph *H* contains only unambiguous causality relations among the factors.

**Table 1:** Ontology mappings created manually by the domain experts

Mapping *m*_*i*_	*O*_1_:*C*_1_	*O*_2_:*C*_2_	r	c
*m*_1_	*O*_1_:Synovial inflammation	*O*_2_:Synovial capsule inflammation	skos:closeMatch	0.75
*m*_2_	*O*_1_:Biochemical imbalance	*O*_2_:Disruption of biochemical balance	skos:relatedMatch	0.5

### Measuring confidence in a hypothesis

Once we obtain the hypothesis graph *H*, we are ready to form the causality hypothesis and perform evidence-based hypothesis testing. Before we delve into this topic, we briefly introduce the notation that we use for the hypothesis graphs throughout this work.

**Notation for hypothesis graphs.** Let *H*=(*N*,*A*) be a directed graph, which we call *hypothesis graph*, with *n*_*i*_∈*N* set of nodes. And *A* is a set of ordered pairs of (*s*,*t*) in *N*, called *arcs*, where *s* denote the *source* of the arc, and *t* the *target* of the arc [54]. A path *π*(*s*,*t*) from *source* node *s* to the *target* node *t* is denoted as *π*_*i*_(*s*,*t*)=(*s*,*n*_*i*_,…,*t*). We write *Π*(*s*,*t*) to denote all possible simple paths in the hypothesis graph from node *s* to the node *t*. A simple path is a path which does not have repeating nodes. And we use $\mathcal {I}(s, t) = \{n_{i} | n_{i} \in \pi _{i}, \forall \pi _{i}(s, t) \in \Pi (s, t)\}$ to refer to all the *interior nodes* which appear in all paths from *s* to *t*.

**Causality hypothesis.** A causal hypothesis asks a question whether some factor (*s*) has caused another factor (*t*). There might be a direct causality relationship from *s* to *t*, or there might exist an indirect causality relationship, such that *s* has caused *t* through some intermediate factors, which might have participated actively or passively to the causality chain from *s* to *t*. These causal chains from *s* to *t* represent different possibilities of how *s* might have caused *t*. We use the notation for hypothesis graph *H* to represent factors as nodes *f*_*i*_∈*N*, direct causality relationships as arcs (*f*_*i*_,*f*_*j*_)∈*E*, and causality chains as paths *Π*(*s*,*t*).

**Fig. 6:**
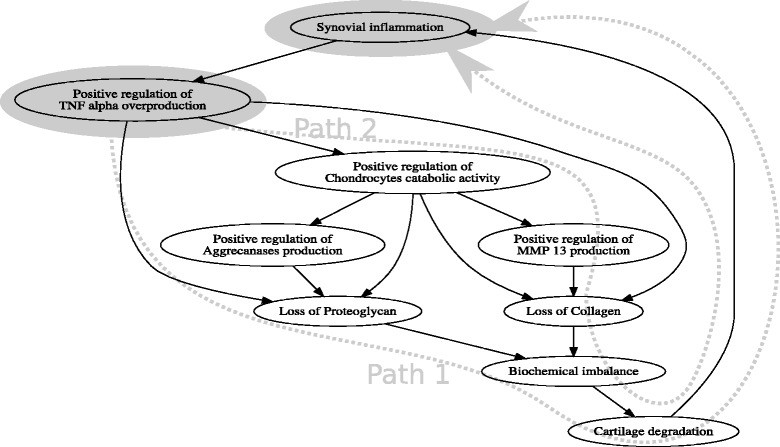
Two possible paths from the factor Positive regulation of TNF alpha overproduction to the factor Synovial inflammation

Consider an example causality hypothesis that postulates that *s*=Positive regulation of TNF alpha overproduction caused *t*=Synovial inflammation in Fig. 6. In our example, we do not have a direct causality relationship between these two factors, however there exist 6 different causal chains, i.e., 6 different ways in which *s* might have caused *t*. In Fig. 6 we present two possible chains of factors (Path 1, Path 2) starting from *s* and leading to *t*.

We are confident in our causality hypothesis – within the domain of the known facts – when we are able to provide evidences to all the factors that participate in causality chains from *s* to *t*. $\mathcal {I}(s, t)$ represents the set of nodes in the hypothesis graph *H*, which correspond to the factors that need to be evidenced, $\mathcal {E}$ is an indicator set which denotes factors evidenced so far, and $\mathcal {C}(s, t, \mathcal {E})$ be the confidence function. Intuitively, confidence in a hypothesis should grow with the number of factors that we are able to evidence, more factors we evidence, more confident we are that *s* did indeed cause *t*. Since, we might have several possibilities of *s* causing *t* we, first, propose to measure confidence of each causality possibility separately, and then, we propose to measure overall causality hypothesis as a sum of the confidences of all the known possibilities (Eq. 1). To this end, our confidence in a causality hypothesis depends on three parameters: i) source of the causality (*s*), ii) target of the causality (*t*), and iii) set of evidenced factors ($\mathcal {E}$).

1 $$ \mathcal{C}_{s}^{t}(\mathcal{E}) = \sum\limits_{\pi \in \Pi(s, t)} \sum\limits_{f \in \pi} \mathcal{F}(f),  $$

Measuring confidence in a causality hypothesis proportionally to the number of evidenced factors might not be correct, there are two sources of uncertainty that might negatively effect our confidence in the hypothesis, even if we collect all the evidences, and should be reflected in the way we measure confidence in the hypothesis: i) the quality of the evidences, i.e., we can surely state that the evidence is not due to errors, and ii) quality of our modelling of the hypothesis. The first source of uncertainty comes from the fact that during our experiments or literature search for the justifications of evidences we might face errors. And the second source of uncertainty comes from the way we model our hypothesis as an assembly of sub-hypotheses, which relies on ontology mappings to merge formalizations of the background knowledge of the hypothesis. During this process we might introduce uncertainty for the matched concepts representing factors of the hypothesis.

To this end, we introduce two functions defined on the nodes of the hypothesis graph, *ϕ*:*N*↦[0…1] that associates weights of the confidence in the ontology mapping to every factor, and represents our confidence in the hypothesis modelling, and *ψ*:*N*↦[0…1] associates weights of the confidence in evidence for each factor. Equation 2 represents the contribution function for the hypothesis factors.

2 $$ \mathcal{F}(f) = \left\{\begin{array}{ll} 0& f \not \in \mathcal{E} \\ & {\text{factor}\, {f}\, \text{not evidenced}} \\ \phi(f) \psi(f) & f \in \mathcal{E} \\ & \text{weighted contribution} \\ & \text{if}\; {f}\, \text{evidenced} \\ \end{array}\right.  $$

**Properties of the confidence function.** Confidence in causality hypothesis is defined as a sum of weighted contributions of factors, that participate in causality possibilities. The contributions of factors is a weighted, and most importantly a non-negative, function (Eq. 1), thus thus as we add more evidenced factors the value of the function, can only grow. Confidence depends on the evidenced factors, it has its minimum value ($\mathcal {C}_{s}^{t}=0$) when we have no evidences ($\mathcal {E}=\emptyset $), and it has its maximum value when all the factors have been evidenced ($argmax \mathcal {C}_{s}^{t} \text {when } \mathcal {E}=\mathcal {I}(s, t))$. To this end, we can normalize our confidence function to the maximum possible confidence value we can obtain, when all the factors have been evidenced, such that the confidence is always measured in the [0…1] range (Eq. 3).

3 $$ 0 = \frac{\mathcal{C}_{s}^{t}(\mathcal{E}=\emptyset)}{\mathcal{C}_{s}^{t}(\mathcal{E}=\mathcal{I})} \le \frac{\mathcal{C}_{s}^{t}(\mathcal{E} \subset \mathcal{I})}{\mathcal{C}_{s}^{t}(\mathcal{E}=\mathcal{I})} < \frac{\mathcal{C}_{s}^{t}(\mathcal{E}=\mathcal{I})}{\mathcal{C}_{s}^{t}(\mathcal{E}=\mathcal{I})} = 1.  $$

## Results

With the help of our domain experts in biology and biomechanical engineering (multi-disciplinary consortium of the EU FP7 “MultiScaleHuman” project [55]) we have been formalizing the background knowledge around factors participating in the process of cartilage degradation, which can be evidenced across different biological scales. This background knowledge has been captured, as a proof of concept, in an OWL 2 ontology *O* and has been iteratively validated with our domain experts. This ontology has been designed to contain a significant amount of axioms which go beyond the usual taxonomical relationships in the biomedical ontologies, and instead, model causality relationships with rich ontology concept construction operators including nested OWL restrictions and property chains. During our interviews (*t*_1_,…,*t*_*n*_) with the domain experts we have been updating the background knowledge formalization ($O_{t_{1}}, \ldots, O_{t_{n}}$), either with the help of our domain experts or by translating discovered causality relationships from the literature ourselves. Each snapshot of the background knowledge $O_{t_{i}}$ has been presented as the results of our methodology of hypothesis graph construction $H_{t_{i}}$ for validation and feedback. To report our results we fix our attention to two specific snapshots of the causality hypothesis, and we refer to them as *H*_*sub*_ and *H*_*broader*_. *H*_*sub*_ has been extracted from the state of the ontology $O_{t_{i}}$, which corresponds to the extent of knowledge of the molecular biologist on causality relationships between the biological processes which lead to cartilage degradation with a focus on cellular and molecular biological scales (*H*_*sub*_ is an equivalent hypothesis graph to what we presented as a normalized hypothesis graph in the “Methods” section). *H*_*broader*_ was extracted from the ontology $O_{t_{j}}$ at time point *t*_*j*_, which corresponds to the ontology $O_{t_{i}}$ updated with more knowledge about factors that lead to cartilage degradation, from organ and behavior biological scales. Table 2 summarizes $O_{t_{i}}, O_{t_{j}}$ with ontology metrics and descriptions, computed with the Protégé ontology editor.

**Table 2:** $O_{t_{i}}, O_{t_{j}}$ ontology metrics

Ontology metric	$O_{t_{i}}$	$O_{t_{j}}$
Axioms	66	151
Logical axiom count	39	92
Declaration axiom count	18	34
Class count	14	30
Object property count	4	4

**Fig. 7:**
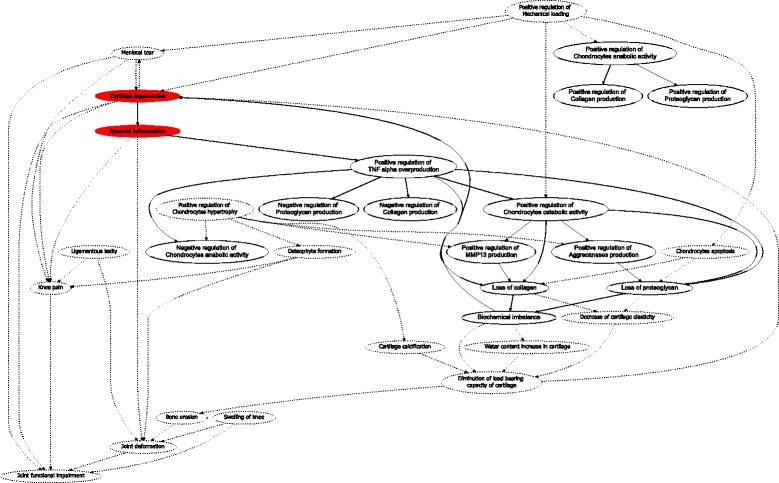
Bold contours show the normalized hypothesis graph “known” to the molecular biologist *H*_*sub*_, whereas the dotted contours delineate the additional knowledge of which the biologist is not aware *H*_*broader*_

In Fig. 7 we notice that *H*_*sub*_=〈*N*_*sub*_,*A*_*sub*_〉 is a subgraph of *H*_*broader*_=〈*N*_*broader*_,*A*_*broader*_〉, such that *N*_*sub*_⊆*N*_*broader*_ and *A*_*sub*_⊆*A*_*broader*_. The additional knowledge (*H*_*broader*_/*H*_*sub*_) is not present in the formalization by the molecular biologist, meaning that he might not be aware about alternative factors that concur during osteoarthritis and might have played a significant role in the causality hypothesis (Fig. 7). The subsequent experiments demonstrate how our methodology supports hypothesis testing by quantifying confidence in a causality hypothesis with incomplete evidences, and provides means to compare confidence measures with different depths of knowledge.

### Robustness of the system in presence of complementary causality relationships

Our methodology is capable of adequately tracking two complementary biological scenarios, where one factor might stand as a cause of two opposite effects. We tested our methodology for hypothesis graph construction with small increments in our knowledge which might lead to big changes in the shape of the causality hypothesis, and what we can understand from it. In particular, at the time point *t*_*i*_ the knowledge on the hypothesis contained causality path from Mechanical loading factor to the Chondrocytes catabolism factor. Indeed, the positive regulation of chondrocytes’ catabolism by mechanical loading has been demonstrated in the literature [56]. However, it is also known that the mechanical loading can also have positive effect on the chondrocytes anabolism (the opposite biological process of catabolism), and thus facilitate proteoglycan and collagen production [57]. Based on the complementary causality effects of mechanical loading on the biochemical balance in cartilage, we can thus hypothesize that mechanical loading might result in both beneficial and detrimental conditions of the joint cartilage. This additional knowledge is reflected in the way our methodology constructs the hypothesis graph. In particular, the normalization patterns (introduced in the Methodology section) split the causality chains starting in mechanical loading, that span two complementary causality possibilities of benign and malign effect on articular joint (Fig. 7). Validly, all the possibilities of mechanical loading leading cartilage degradation pass through the factor positive regulation of chondrocytes catabolism and we do not have a situation where mechanical loading leads to cartilage degradation by passing through positive regulation of chondrocytes anabolism. Conversely all the causality chains which lead from mechanical loading to collagen or proteoglycan production pass through chondrocytes anabolism factor.

### Relative confidence measurement

This experiment demonstrates how molecular objectives can measure his confidence in the causality hypothesis according to his knowledge on causality relationships (*H*_*sub*_) and can compare it to the confidence measure when we add more knowledge *H*_*broader*_. We simulate the case where the molecular biologist wants to test a hypothesis that *s*=Synovial inflammation has caused *t*=Cartilage degradation. We treat *H*_*broader*_ as a coarse approximation of our universal knowledge on all possible causalities which lead from *s* to *t*, and *H*_*sub*_ as a personal view of that universal knowledge by the molecular biologist.

Table 3 summarizes network statistics of the two graphs. In particular, in the universal hypothesis graph *H*_*broader*_ there are 24 possible causal chains which lead from *s* to *t*, whereas in the subgraph *H*_*sub*_ we have only 6 possible causal chains, which means that the molecular biologist is missing a significant amount of knowledge about the causalities that he is studying. Moreover, in the universal knowledge of causality hypothesis we have 12 ($|\mathcal {I}_{H_{broader}}| = 12$) factors that can potentially be evidenced and would contribute positively to the overall confidence of the hypothesis, whereas in the restricted knowledge case we are aware of only 9 ($|\mathcal {I}_{H_{sub}}| = 9$) factors which need to be evidenced to obtain the maximum confidence in the same hypothesis that *s* has caused *t*. To study the behavior of the confidence function $C_{s}^{t}$ in these two cases we perform the following tests: i) study the evolution of the confidence function separately for two graphs, ii) normalize the confidence function with the maximum possible confidence for individual graphs, iii) normalize the two confidence functions with the maximum confidence in the universal graph. Note that, the parameter for the confidence function is the set of evidenced nodes, where each node may have different importance value, as defined by the weighting function $\mathcal {F}$. To take into account all the possible variability of the confidence function we compute the distributions of the confidence values for a gradually increasing number of evidences. That is, we start with the case where the evidence set is empty, corresponding to the initial phase of hypothesis testing and where our confidence is 0. Then, we compute the distribution of confidences for all evidence sets of size (cardinality) 1, corresponding to different choices of choosing one factor to evidence. For instance, for the universal hypothesis graph *H*_*broader*_ we have 12 ways to to prove hypothesis by evidencing only one factor (out of 12 possible), whereas for *H*_*sub*_ we have 9 factors to choose from. We continue computing confidence distributions until we reach the full evidence set.

**Table 3:** Statistics of the graphs

Statistic	*H*_*sub*_	*H*_*broader*_
Number of nodes |*N*|	15	30
Number of arcs |*A*|	19	57
Number of possible causal chains from *s* to *t*	6	24
Number of possible factors to evidence $| \mathcal {I} |$	9	12

**Fig. 8:**
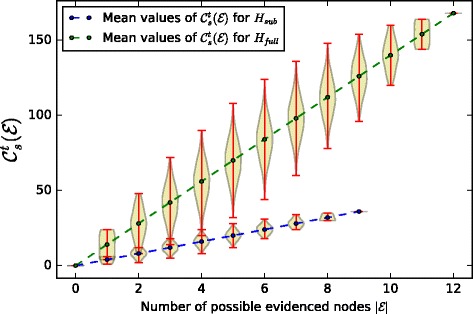
Confidence distributions for gradually increasing sizes of evidence sets for the two graphs *H*_*sub*_,*H*_*broader*_, with a trivial weighting function $\mathcal {F}(f) = 1$

Figure 8 represents the distribution of confidences computed with $C_{s}^{t}$ (Eq. 1) for gradually increasing sizes of evidence sets, with a trivial weighting function of factors– $\mathcal {F} = const \, 1$ – where every factor has equal contribution to the causality chains. The mean values of the confidence distributions grow linearly as we increase the number of evidences, as expected, the maximum confidence value obtained in the universal case is bigger than in the restricted case because we take into account more possibilities in the universal case. We now use the individual maximum mean confidence values for each graph to scale our distributions, such that they always stay in the 0..1 range.

**Fig. 9:**
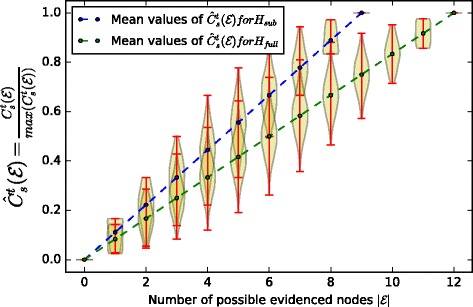
Confidence distributions for gradually increasing sizes of evidence sets for the two graphs *H*_*sub*_,*H*_*broader*_, normalized by its maximum possible confidence value

Figure 9 shows the normalized version of the confidence distributions, namely $\hat {C}_{s}^{t} = \frac {C_{s}^{t}}{max(C_{s}^{t})}$ for *H*_*sub*_ and *H*_*broader*_. In particular, it shows that a molecular biologist, relative to his extent of knowledge, obtains the 100% confidence in his causality hypothesis by evidencing all the possible factors which contribute to all the possible ways in which *s* might have caused *t*, however, with the same amount of evidence, but taking into account universal knowledge about the causality possibilities, his confidence is less than 100%, which shows that he has missed some important causality possibilities. To quantify this uncertainty, which is proportionate to the amount of missed causality possibilities, we scale both confidence distributions by the maximum confidence value that we may obtain in the universal case.

**Fig. 10:**
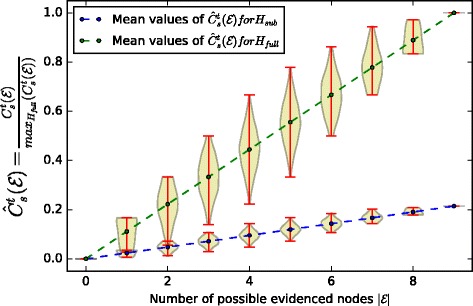
Confidence distributions for gradually increasing sizes of evidence sets for the two graphs *H*_*sub*_,*H*_*broader*_, normalized by the maximum possible confidence value in the universal case

Figure 10 demonstrates the *relative confidence* of the molecular biologist to the universal causality hypothesis for the same evidenced sets. The x-axis is truncated to evidence sets of size 9, since molecular biologist is only aware of 9 factors which need to be evidenced to prove his hypothesis. If we collect the mean values of the confidence distributions in two vectors *x*_1_,*x*_2_ then we can quantify the error as their Euclidean distance ∥*x*_1_−*x*_2_∥. In Table 4 we summarize the errors which quantify the uncertainty in obtained confidence measures with respect to the universal case for different weighting functions $\mathcal {F}_{i}$. These weighting functions were chosen as follows: i) $\mathcal {F}_{1}$ trivial weighting of importance of factors, ii) $\mathcal {F}_{2}$ random weighting of importance of each factor, iii) $\mathcal {F}_{3}$ gives more importance to factors which molecular biologist is aware of, whereas those that he is not aware of are given less importance, iv) $\mathcal {F}_{4}$ opposite to $\mathcal {F}_{3}$, we give more importance to factors that molecular biologist is not aware of and we decrease the importance of factors that he is aware of. The error variation is intuitive, if we evidence the most important factors, even if we miss other factors and other causality chains, but whose importance to the overall hypothesis is significantly smaller, then we are more *confident* even with a restricted knowledge of the causality possibilities. Vice-versa, if we evidence less important factors and we miss the important ones, then our confidence is much more compromised.

**Table 4:** Mean squared error between the confidence distributions for different weighting functions $\mathcal {F}$

Weighting function $\mathcal {F}_{i}$	Error
$\mathcal {F}_{1}(f) = 1$	2.17
$\mathcal {F}_{2}(f) = random(0, 1)$	2.09
$\mathcal {F}_{3}(f) = 1$ if $f \in I_{H_{sub}}$, otherwise 0.1	1.95
$\mathcal {F}_{4}(f) = 1$ if $f \in I_{H_{broader}}$, otherwise 0.1	2.96

### Local importance of factors

Importance of the factors for a causality hypothesis can be deduced from our confidence measure defined on the hypothesis graph. The factors ranked as the most important may help the researchers prioritize their next experiments, studies, and may help in the discovery of the potential collaborations with other scientists. Analogously, the factors that are identified as the least important for a specific causality hypothesis hint on the lack of knowledge about the possibly missing causality relationships, and might represent an opportunity to focus on an underresearched topic. In particular, $C_{s}^{t}$ measures our confidence in the causality hypothesis that factor *s* caused *t* with a given set of evidenced nodes $\mathcal {E}$. This function accumulates the weighted contribution of all evidenced nodes in each causality possibility leading from *s* to *t*. When we first start proving our hypothesis we do not have any evidence and we have a choice of $\mathcal {I}$ to evidence from. However, do we need to evidence all the factors in the interior of the causality hypothesis $\mathcal {I}$? What if we can only obtain an incomplete set of evidences, which factors should we choose? Intuitively, we should first focus on evidencing factors which are most important in our causality hypothesis. But how can we assess the importance of each factor in the causality hypothesis? In this experiment, we propose a general approach to assessing the local importance of factors, independently of the weighting function $\mathcal {F}$. To do so we start with a case where we do not have any evidence $\mathcal {E} = \emptyset $, we then rank each factor *f*_*i*_ in the causality hypothesis by its potential contribution to the confidence in the causality hypothesis if it was evidenced $|\mathcal {C}_{s}^{t}(\mathcal {E} \cup f_{i}) - \mathcal {C}_{s}^{t}(\mathcal {E} = \emptyset)|$.

**Fig. 11:**
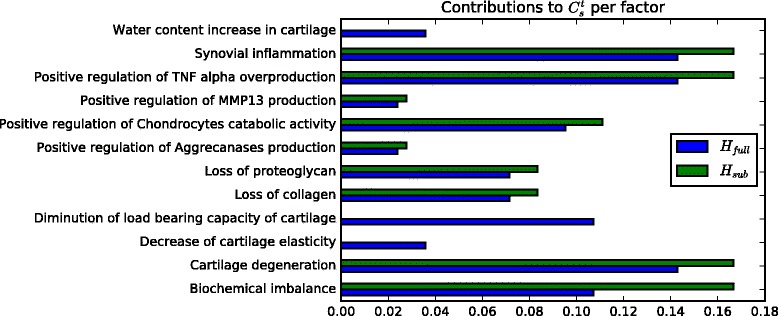
Contributions of the interior factors of the hypothesis *s* caused *t* for two hypothesis graphs *H*_*sub*_,*H*_*broader*_ with two different depths of knowledge

Figure 11 depicts the variation of potential contributions to the overall confidence measure $C_{s}^{t}$ for each factor *f*_*i*_. In particular, we can observe that in both cases: *H*_*sub*_ restricted personal view of the hypothesis, and *H*_*broader*_ universal causality hypothesis the most important factors are: Positive regulation of TNF alpha overproduction, *s*=Synovial inflammation, *t*=Cartilage degeneration and Biochemical imbalance. Indeed, to prove that *s* has resulted in *t* our best strategy is to focus on evidencing those two factors, however, given our knowledge of causality relationships, we might choose to evidence alternative factors to obtain the same overall confidence in the validity of our causality hypothesis. We also observe that by extracting more knowledge on causality relationships more important factors to our causality hypothesis emerge, i.e., the factors which we did not know about before. For instance, Decrease of cartilage elasticity and Water content increase in cartilage have relatively low potential confidence contributions (< 0.04) and thus our unawareness of the contribution to causality hypothesis of these factors is not so penalizing. Yet, Diminution of load bearing capacity of cartilage is capable of contributing more than 10% of the overall confidence measure $C_{s}^{t}$. It is also interesting to observe that adding knowledge (*H*_*broader*_) reduces the importance of Biochemical imbalance factor to the point that it is no longer one of the most important factors in the causality hypothesis.

### Generalization of the hypothesis configuration

In the previous experiment we identified the most important factors, such that evidencing them would maximize our confidence in the causality hypothesis that *s* resulted in *t*. We can use the local importance of factors to the hypothesis configuration to target our evidence collection. Suppose we managed to evidence the four most important factors for the hypothesis graph *H*_*sub*_, which we summarize in Table 5.

**Table 5:** 4 Most important factors for *H*_*sub*_ in the two hypothesis graphs and their relative confidence values in both *H*_*sub*_ and *H*_*broader*_

Evidence set $\mathcal {E}_{sub}$	Importance for *H*_*broader*_	Importance for *H*_*sub*_
Biochemical imbalance	0.10	0.16
Cartilage degeneration	0.14	0.16
Positive regulation of TNF alpha overproduction	0.14	0.16
Synovial inflammation	0.14	0.16
	$C_{s}^{t} (\mathcal {E}_{sub})$ for *H*_*broader*_	$C_{s}^{t}(\mathcal {E}_{sub})$ for *H*_*sub*_
	0.53	0.66

For the same evidence set $\mathcal {E}_{sub}$ we obtain the normalized confidence of $C_{s}^{t}=0.66$ for *H*_*sub*_ and $C_{s}^{t}=0.53$ for *H*_*broader*_. Now, we ask ourselves a question “with the same evidence set what other causalities can we prove (with the same confidence)?”. If we keep the same evidence set $\mathcal {E}_{sub}$ we are able to prove causalities with a confidence > 60*%* as depicted in Table 6. These causalities correspond to very similar causality chains, as our initial causality hypothesis that Synovial inflammation has results in Cartilage degradation.

**Table 6:** Other causalities we can prove (> 60*%* confidence) with the same evidence set $\mathcal {E}_{sub}$

Source *s*	Target *t*	$C_{s}^{t} (\mathcal {E}_{sub})$ for *H*_*broader*_	$C_{s}^{t} (\mathcal {E}_{sub})$ for *H*_*sub*_
Cartilage	Biochemical imbalance	0.66	0.66
degeneration	Negative regulation of Collagen production	0.75	0.75
	Positive regulation of TNF alpha overproduction	1.00	1.00
Loss of	Positive regulation of TNF alpha overproduction	0.62	0.80
collagen			
Loss of	Positive regulation of TNF alpha overproduction	0.62	0.80
proteoglycan			
Synovial	Negative regulation of Chondrocytes anabolic activity	0.66	0.66
inflammation			
	Negative regulation of Collagen production	0.66	0.66
	Negative regulation of Proteoglycan production	0.66	0.66
	Positive regulation of Chondrocytes catabolic activity	0.66	0.66
	Positive regulation of TNF alpha overproduction	1.00	1.00

Intuitively, Table 7 demonstrates that for the same evidence set, as we add more knowledge (*H*_*broader*_) we are able to prove more causality relationships, with a good confidence (> 50*%*).

**Table 7:** Causalities we can prove (> 50*%* confidence), as we add more knowledge, and which we cannot prove with our restricted knowledge of causality relationships

Source *s*	Target *t*	$C_{s}^{t} (\mathcal {E}_{sub})$ for *H*_*broader*_	$C_{s}^{t} (\mathcal {E}_{sub})$ for *H*_*sub*_
Cartilage calcification	Positive regulation of TNF	0.60	0.0
	alpha overproduction		
Diminution of load bearing	Biochemical imbalance	0.57	0.0
capacity of cartilage	Negative regulation of	0.60	0.0
	Chondrocytes anabolic activity		
	Negative regulation of	0.60	0.0
	Collagen production		
	Negative regulation of	0.60	0.0
	Proteoglycan production		
	Positive regulation of Chondrocytes	0.60	0.0
	catabolic activity		
	Positive regulation of TNF	0.75	0.0
	alpha overproduction		
	Synovial inflammation	0.66	0.0
Meniscal tear	Biochemical imbalance	0.57	0.0
	Negative regulation of	0.60	0.0
	Collagen production		
	Negative regulation of	0.60	0.0
	Proteoglycan production		
	Positive regulation of Chondrocytes	0.60	0.0
	catabolic activity		
	Positive regulation of TNF	0.75	0.0
	alpha overproduction		
Water content increase	Positive regulation of TNF	0.60	0.0
in cartilage	alpha overproduction		

Generalization of the hypothesis configuration leads to the scenarios where the seemingly wrong causality relationships, might actually be explained with plausible interpretations. One such example scenario is when we obtain the significant confidence (0.60) in a causality hypothesis that Cartilage calcification might result in Positive regulation of TNF alpha overproduction (line 1 in Table 7). First, it is tempting to say that this is a wrong hypothesis, and is due to the error in the formalization of the background knowledge on causality relationships. Partly, because calcification of cartilage entails cell apoptosis and thus should cause the decrease of levels of TNF alpha cytokine cells. However, we get the high confidence score in this causality due to the presence of a path from Cartilage calcification to Positive regulation of TNF alpha overproduction (see Fig. 7). This path represents our knowledge that calcified cartilage will result in degeneration of cartilage tissue, which will provoke synovial inflammation, and we hypothesized that synovial inflammation will result in positive regulation of TNF alpha. After a discussion with our domain experts we reached the conclusion that, although this causality relationship between calcified cartilage and positive regulation of TNF alpha might seem contradictory, there actually might be a plausible explanation. Namely, while the calcification causes tissue death in cartilage, it does so only in a specific region of cartilage. The calcified region, however, will induce the diminution of the load bearing properties of the whole cartilage, and this will provoke the synovial inflammation, which, in turn, will result in excessive levels of TNF alpha in the neighbouring regions of the cartilage (neighbouring to the calcified region).

### Prototype

**Fig. 12:**
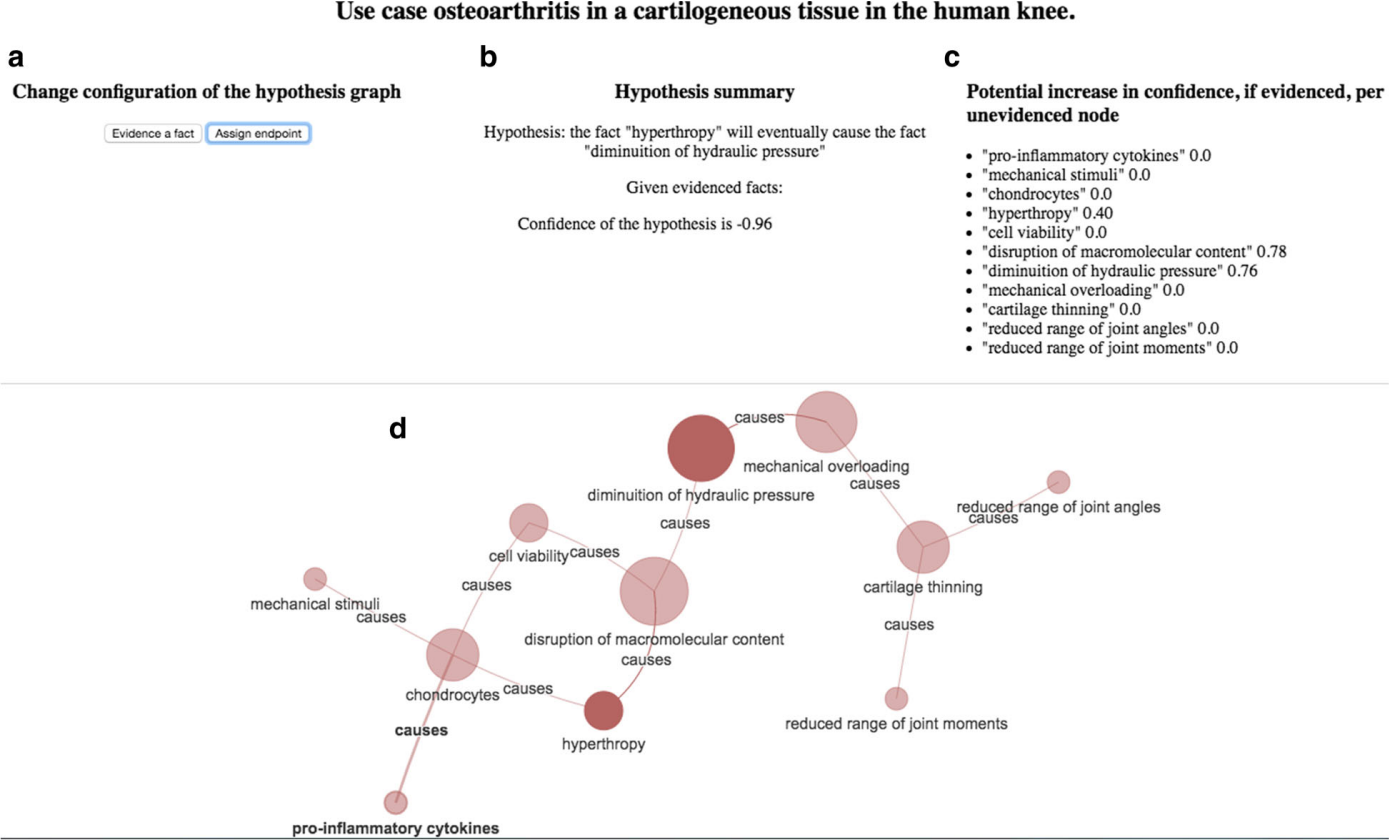
The interface of the prototype is divided into 4 logical blocks: **a**) control over the hypothesis configuration *h*, **b**) hypothesis summary, **c**) local importance of nodes in the hypothesis and **d**) visualization of the hypothesis graph

We implemented a prototype (Fig. 12) to interactively apply and present the proposed methodology for causality hypothesis testing on the obtained hypothesis graphs. The demo of the prototype is available at http://hypothtest.plumdeq.xyz/test/. Source code for the hypothesis testing of the prototype and proof of concept ontologies, as well as the Jupyter Notebooks (reproducible experiments presented in this manuscript) are available on GitHub at https://github.com/plumdeq/hypothtest (see “Availability of data and materials” subsection).

The interface of the prototype is divided into 4 logical blocks, labeled *a, b, c, d* in Fig. 12.

**(A) Control over the hypothesis configuration.** The users can change the hypothesis configuration in two modes - i) identifying the boundary nodes *s*,*t*, ii) selecting the evidenced nodes $\mathcal {E}$. Each mode is triggered by clicking on an associated button (see Fig. 12a), and then selecting the specific nodes in the hypothesis graph (Fig. 12d).

**(B) Hypothesis summary.** A textual summary of a current hypothesis configuration (see Fig. 12b).

**(C) Local importance of nodes in the hypothesis.** Local importance of each node with respect to the hypothesis configuration.

**(D) Visualisation of the hypothesis graph.** Interactive network visualisation with the force directed layout [58] of the hypothesis graph *H*. The users can interactively click on the nodes and drag them for a visually better spatial distribution of the network. The boundary nodes are visually distinguished as completely *opaque* nodes in the hypothesis graph (Fig. 12), while all other nodes are semi-opaque. Evidenced nodes are visually distinguished as *green* nodes. Consequently, if a node *n*_*i*_ is both *evidenced* and either a *source* or a *target* of the confidence evaluation, then it will be *opaque green*. The backend (server) of the prototype constructs hypothesis graphs, computes importance measures on each node of the graph, and evaluates confidence in the hypothesis configuration. The frontend (client) is responsible for the interactive visualisation of the hypothesis graph, and serves as a user interface. In particular the user can interactively assign the boundary nodes, and mark nodes as evidenced. The user input is then transmitted to the backend via custom data exchange protocol, based on JSON files. Each time the user changes the configuration of the hypothesis (i.e., evidences/unevidences node or assigns new source or target nodes of the confidence evaluation the hypothesis confidence is reevaluated and the results are sent back to the client.

## Discussion

We evaluated our methodology on a hypothesis graph which covers our use-case scenario of cartilage degradation during osteoarthritis. The obtained hypothesis graph represents both contributing factors which may cause cartilage degradation and the factors which might be caused by the cartilage degradation. Hypothesis graph construction (see “Robustness of the system in presence of complementary causality relationships” section) has proven to be robust to the addition of potentially contradictory information on the simultaneously positive and negative effects, by adequately separating two complementary causality scenarios. By evaluating our methodology for relative confidence measurement (see “Relative confidence measurement” section) we have observed the following: i) the more evidences we are able to provide (as $\mathcal {E} \to \mathcal {I}$) the bigger is our overall confidence function (confidence grows $\mathcal {C}_{s}^{t} \uparrow $), ii) our relative confidence to the universal knowledge of the hypothesis (i.e., the difference in confidences) is proportionate to how much knowledge on causal possibilities we lack with respect to the universal causality hypothesis, the less causality possibilities we take into account in our formalization the smaller is our confidence in the causality hypothesis with respect to the universal knowledge of the causality hypothesis, iii) our confidence in the causality hypothesis increases when we evidence more factors favored by $\mathcal {F}$ with respect to the universal formalization of the causality hypothesis, even if we do not have full knowledge of the causality possibilities. The domain experts found that our computational methodology for assessing confidence in a causality hypothesis proportionally to the amount of available knowledge, corresponds to their subjective assessments of confidences in an investigated hypothesis. Moreover, the obtained confidence measures for the specific causality hypotheses have been validated by our domain experts, and, in some cases, have led to new interpretations of the already known causality connections (see “Generalization of the hypothesis configuration” section).

### Limits, assumptions and dependencies of methodology.

Overall our framework is dependant on the validity, quality and the richness of the modelling, which will induce the final shape and topology of the hypothesis graph and the way the confidence is assessed by using our methodology for confidence assessment. Of course, our methodology has its limits and has its assumptions and dependencies. Main assumptions and dependencies of the methodology for hypothesis testing rely on: i) ontological commitment of the input ontologies *O*_*i*_ that formalize background biological knowledge on causality relationships, ii) biological validity and logical consistency of the formalized knowledge - input to the framework, iii) weighting scheme of factors of the hypothesis that measure the quality of the ontology matching of concepts used to assemble the final ontology, and the confidence of the obtained evidence for a specific factor *f*_*i*_. Ontological commitment of the modelled realities representing causality relationships among the factors should follow the good design patterns for modelling causalities, for both concepts and relationships that interrelate those concepts. In particular, we consider the processual perspective of a *disease* as a causal chain structure as in River Flow Model of Diseases [25] as opposed to an object-like perspective of a whole constituting a disease as in Ontology of General Medical Sciences (OGMS) [59]. As has been argued by Rovetto and Mizgouchi [25], the causality in OGMS is unstated, implicit or stated indirectly. The general account of disease in OGMS draws ideas from Scheuermann et al. [60], and distinguishes diseases from disease courses. Diseases in OGMS are treated as dispositions potentially realizable via pathological processes, and have some disorders as their physical basis. In our work, we focus on causality relationships which constitute a disease course, and reason on these relationships by relying on graph analysis techniques. Due to this modelling choice we expect the input ontologies to follow the RFM account of disease as a causal chain structure. Specifically, our methodology for hypothesis graph construction extracts causality relationships from the assembled ontology such that the final hypothesis graph contains nodes as occurrents, either biological processes, as exemplary modelled in the Gene Ontology [20], or as conditions (abnormal states), according to the guidelines of the RFM. The causality relationships should be compliant with the Relation Ontology [24], which, among other types, covers concurrent and overlapping causality relationships between the occurrent entities, relying on Allen interval algebra calculus for temporal logic [61]. Strategies toward harmonization between disease accounts in OGMS and RFM are brought up in Rovetto and Mizgouchi [25]. Hypothesis graph creation with input ontologies following the OGMS modelling of disease could represent a promising future direction for the community.

Weighting scheme for the factors of the hypothesis graph will largely depend on the context (e.g., studied disease), the quality of the ontology mappings, and the confidence of the obtained evidence. Mappings to guide the assembly (i.e., link factors from different hypothesis) can be discovered in online resources like UMLS Metathesaurus [49] or BioPortal [50, 51], or using state of the art ontology alignment systems like LogMap [52] or AML [53]. Confidence in the obtained evidence will depend on the methodology of the experiment and should be assessed by the executioner of the experiment, which might entail subjective importance weight of the factor and might have subjective consequences on the computation of the overall confidence in the causality hypothesis with our framework.

## Conclusions

We have presented a promising and nascent methodology for the translation of biological knowledge on causality relationships of biological processes and their effects on conditions to a computational framework for shared hypothesis testing. Furthermore, we have defined a knowledge-driven, and evidenced-based way of measuring confidence in a causality hypothesis proportionally to the amount of available knowledge and collected evidences. The methodology resumes in two points: hypothesis graph construction from the formalizations of the background knowledge on causality relationships, and confidence measurement in a causality hypothesis as a normalized weighted path computation in the hypothesis graph. Lastly, we have made the source code and materials available to the community on GitHub at https://github.com/plumdeq/hypothtest (see “Availability of data and materials” subsection).

Herein we took advantage of our domain experts to build a simplified and a tractable version of a causality hypothesis graph of cartilage degradation during to osteoarthritis, and to validate our methodology for confidence assessment of causality hypothesis. The evaluation results, the feedback from our experts, and the lessons learnt from this overall experience allow us to conclude that a methodology for shared hypothesis testing could be incorporated as an invaluable asset to the online biological knowledge graph mining services. In particular, our hypothesis graph construction methodology could be used routinely to enrich biological knowledge graphs (e.g., Knowledge Bio [62]) and online databases (e.g., Gene Wiki [63]) by extracting the causality relationships information from OWL 2 ontologies. Of course, the proposed set of patterns for the normalization of the hypothesis graph will have to be augmented and tuned for a specific studied context. We, for instance, defined graph rewriting normalization patterns to deal with complementary biological scenarios of simultaneously positive and negative regulations of biological processes (see “Robustness of the system in presence of complementary causality relationships” section). In fact, the graph rewriting patterns is a general paradigm for the transformation of formalized knowledge on a specific biological pattern into its equivalent graph representation and might open an opportunity for more research and practical contributions from the biomedical community.

Shared hypothesis testing services built on top of the confidence measurement (see “Relative confidence measurement” section), and the inference procedures it induces (see “Generalization of the hypothesis configuration” section), will enhance the biological knowledge graphs with advanced simulation functionalities for continuous research. These services could support researchers in literature review for their experimental studies, planning and prioritizing evidence collection acquisition procedures, and testing their hypotheses with different depths of knowledge on causal dependencies of biological processes and their effects on the observed conditions. Measuring confidence in a causality hypothesis relatively to the already discovered causality relationships might serve in the assessment of the fairness of the obtained results, and its significance to the already known results. We believe that the shared hypothesis testing could serve as an important asset for the costless re-enactment of the experiments, and might eventually contribute to the future, purely computational benchmarks for the validation of the experiments.

## Abbreviations

ABox: Description logics ground sentences stating where in the hierarchy individuals belong
DL: Description logics
FMA: Foundational model of anatomy ontology
MRI: Magnetic resonance image
OA: Osteoarthritis
OWL: Web ontology language
RDF: Resource description framework
SNA: Social network analysis

## Footnotes

1 See https://www.bioontology.org/wiki/index.php/BioPortal_Mappings
